# Power Production and Biochemical Markers of Metabolic Stress and Muscle Damage Following a Single Bout of Short-Sprint and Heavy Strength Exercise in Well-Trained Cyclists

**DOI:** 10.3389/fphys.2018.00155

**Published:** 2018-03-05

**Authors:** Morten Kristoffersen, Øyvind Sandbakk, Espen Tønnessen, Ida Svendsen, Gøran Paulsen, Elisabeth Ersvær, Irene Nygård, Kari Rostad, Anita Ryningen, Vegard V. Iversen, Knut Skovereng, Bent R. Rønnestad, Hilde Gundersen

**Affiliations:** ^1^Department of Sport and Physical Activity, Western Norway University of Applied Sciences, Bergen, Norway; ^2^Department of Neuroscience and Movement Science, Centre for Elite Sports Research, Norwegian University of Science and Technology, Trondheim, Norway; ^3^Norwegian Olympic Federation, Oslo, Norway; ^4^Faculty of Engineering and Science, Western Norway University of Applied Sciences, Bergen, Norway; ^5^Section for Sport Science, Inland Norway University of Applied Sciences, Lillehammer, Norway

**Keywords:** strength training, short-sprint training, recovery, cycling, biochemical markers, power production

## Abstract

**Purpose:** Although strength and sprint training are widely used methods in competitive cycling, no previous studies have compared the acute responses and recovery rates following such sessions among highly trained cyclists. The primary aim of the current study was to compare power production and biochemical markers of metabolic stress and muscle damage following a session of heavy strength (HS) and short-sprint training (SS).

**Methods:** Eleven well-trained male cyclists (18 ± 2 years with maximal oxygen uptake of 67.2 ± 5.0 mL·kg^−1^·min^−1^) completed one HS session and one SS session in a randomized order, separated by 48 h. Power production and biochemical variables were measured at baseline and at different time points during the first 45 h post exercise.

**Results:** Lactate and human growth hormone were higher 5 min, 30 min and 1 h post the SS compared to the HS session (all *p* ≤ 0.019). Myoglobin was higher following the HS than the SS session 5 min, 30 min and 1 h post exercise (all *p* ≤ 0.005), while creatine kinase (CK) was higher following the HS session 21 and 45 h post exercise (*p* ≤ 0.038). Counter movement jump and power production during 4 sec sprint returned to baseline levels at 23 and 47 h with no difference between the HS and SS session, whereas the delayed muscle soreness score was higher 45 h following the HS compared to the SS session (*p* = 0.010).

**Conclusion:** Our findings indicate that SS training provides greater metabolic stress than HS training, whereas HS training leads to more muscle damage compared to that caused by SS training. The ability to produce power remained back to baseline already 23 h after both training sessions, indicating maintained performance levels although higher CK level and muscle soreness were present 45 h post the HS training session.

## Introduction

Road cycling is an endurance sport with competitions typically lasting several hours (Coyle, 1999; Jeukendrup et al., 2000; Padilla et al., 2000; Faria et al., 2005). However, many races are decided in a sprint finish (Martin et al., 2007) where high power over a short period (~10 s) of time is critical. To improve sprint power in cycling, many competitive cyclists regularly supplement their endurance training with heavy strength (HS) and/or short-sprint (SS) training. These strategies are supported by previous studies, showing positive effects of both HS training (Rønnestad et al., 2010, 2015, 2016; Aagaard et al., 2011; Vikmoen et al., 2016) and SS training (Creer et al., 2004; Sloth et al., 2013; Hebisz et al., 2016) on overall cycling performance and aerobic endurance indices in well-trained cyclists.

HS and SS exercise exert different loads on the neuromuscular and metabolic systems (Coffey et al., 2009). While HS is normally executed with high loads and slow concentric and eccentric muscle actions (Kraemer and Ratamess, 2004), SS in cycling is done one the bike and involves mainly concentric muscle work with lower loads and higher velocity contractions (Martin et al., 2007). Although this reasoning implies that both acute responses and recovery rates following HS and SS sessions should differ, the current studies investigating such responses have been conducted on athletes performing either high intensity or strength training, or on untrained and less trained subjects (Barnett, 2006). However, an athlete's training status would have a significant impact on both acute responses and recovery rates (Brancaccio et al., 2007, 2010; Bishop et al., 2008) highlighting the importance of conducting such studies on highly-trained participants.

Understanding training load and recovery in a given sport is imperative when designing training programs, because these variables determines long-term adaptations (Bishop et al., 2008). As a measure of external workload, power output is commonly used both in cycling and in strength training, whereas internal training load is typically estimated based on physiological and perceptual responses such as oxygen uptake, heart rate (HR), blood lactate concentration ([La^−^]) (Borresen and Lambert, 2009), and rating of perceived exertion (RPE) (Borg, 1982; Wallace et al., 2009). The changes in these variables are also used to measure recovery status following HS training, in combination with variations in strength and power performance (Raastad and Hallen, 2000; Andersson et al., 2008; Haugvad et al., 2014) and muscle soreness (Armstrong, 1984; Nosaka et al., 2002). Based on such measurements, HS training programs containing 2–4 sets of 4–8 repetitions are shown to require 24–72 h of recovery in well-trained athletes (Paulsen et al., 2012). On the other hand, recovery following SS sessions has not yet been investigated, and possible differences in recovery rates following HS and SS training are therefore not clear. However, the acute responses of [La^−^] and hGH seems to be higher after high intensity training and sprint training compared to HS training (Kraemer et al., 1990; Godfrey et al., 2003; Stokes et al., 2004), and Mb and CK levels are showed to be higher after HS training (Brancaccio et al., 2007, 2010; Speranza et al., 2007).

In addition to power production and perceptual measurements, various blood (biochemical) markers may provide a more detailed picture of how the various systems are loaded during a training session, as well as the subsequent rate of recovery (Brancaccio et al., 2010; Bessa et al., 2016). For example, creatine kinase (CK) (Koch et al., 2014) and myoglobin (Mb) (Speranza et al., 2007; Soares and Bozza, 2016) provide an indication of muscle damage, while human growth hormone (hGH) and [La^−^] during and immediately after exercise (Smilios et al., 2003; Gladden, 2004; Stokes et al., 2004) are regarded markers of the metabolic disturbances following training sessions.

The primary aim of the current study was to compare power production and biochemical markers of metabolic stress and muscle damage following a HS and a SS training session, as well as the 45-h recovery rates in well-trained cyclists. The secondary aim was to compare the changes in these values compared to their baseline levels. We hypothesized that biochemical indicators of muscle damage recover more slowly after HS compared to SS training, whereas acute metabolic responses are altered to a greater extent after SS training in well-trained cyclist.

## Materials and methods

### Participants

Twelve well-trained male cyclists gave their written, informed consent to participate in the study. All cyclists had experience with HS and SS training from their daily training. To be included, the following criteria was fulfilled: (1) competitive cycling at national or international level, (2) maximal oxygen uptake ($\overset{\cdot }{V}$O_2max_) of ≥60 mL·kg^−1^·min^−1^, (3) implemented strength training including squat, hip flexion and leg press twice a week for a minimum of 4 weeks before testing, and (4) currently healthy and free from injury. One participant was excluded from the study due to illness. Baseline characteristics of the participants are presented in Table 1. The Regional Committee for Medical and Health Research Ethics in West Norway evaluated our study not to include any medical or health related ethical concerns, and the study was then approved by The Norwegian Data protection Authority.

**Table 1:** Physiological characteristics of the male cyclists included in the study (*n* = 11).

Age (years)	18 ± 2
Body height (cm)	181 ± 7
Body mass (kg)	71 ± 5
Body fat (%)	11 ± 3
$\overset{\cdot }{V}$O_2*max*_ (mL·kg^−1^·min^−1^)	67.2 ± 5.0
$\overset{\cdot }{V}$O_2*max*_ (L·min^−1^)	4.8 ± 0.4
Maximal aerobic power (W)^*^	401 ± 39
Power output at [La^−^] of 4 mmol·L^−1^ (W)	287 ± 30
Power output at [La^−^] of 4 mmol·L^−1^ (W·kg^−1^)	4.1 ± 0.4

* *The highest average of two consecutive 30 s measurements, [La^−^]: Blood lactate concentration*.

### Overall design

The study was completed with a crossover design. Each participant completed either a HS or a SS session in a randomized order. There were 48 h between the two sessions for all participants. HR, RPE, power, and [La^−1^] were measured during each of the training sessions. Biochemical variables were measured before breakfast (baseline) and 5 min, 30 min, 1 h, 21 h and 45 h post sessions. Power production was measured at baseline (after breakfast) as well as 23 h (CMJ and 4-sec sprint test) and 47 h (CMJ) post sessions. RPE was measured 0 min, 30 min and 1 h post sessions, while delayed muscle soreness (DOMS) was measured 21 h and 45 h post sessions.

### Preliminary testing

Approximately 14 days before the first experimental training session, the participants completed submaximal and maximal testing on a cycle ergometer (Lode Excalibur Sport, Groningen, Netherlands). The submaximal testing was performed with stepwise increase in workload every 5 min, starting with 5-min at 125 W and increase of 50 W until a capillary blood lactate concentration ([La^−^]) of 2 mmol·L^−1^, followed by increase of 25 W until a [La^−^] of 4 mmol·L^−1^ or higher. HR was recorded (Polar V800, Kempele, Finland) during the final min of each stage, a fingertip blood sample was collected during the final 30 s for analysis of [La^−1^] (Biosen S-line, EKF diagnostics, Germany). Power output at [La^−^] of 4 mmol·L^−1^ was calculated from the relationship between [La^−^] and power output, using linear regression between data points.

After the submaximal test, each participant cycled at low intensity for 10 min before a continuous, incremental cycle ergometer test to volitional exhaustion determined $\overset{\cdot }{V}$O_2max_. The test began one stage below the workload that elicited [La^−^] of 4 mmol·L^−1^ in the submaximal test, with increments of 25 W every minute. HR was measured continuously throughout the test, and the peak value recorded was defined as HR_max_. Expired gas was collected and analyzed continuously using a computerized metabolic system with mixing chamber (Oxycon Pro, Erich Jaeger GmbH, Hoechberg, Germany), calibrated before every test with certified calibration gases of known concentrations and a 3-L calibration syringe (CareFusion, Hoechberg, Germany). The determination of maximal $\overset{\cdot }{V}$E, $\overset{\cdot }{V}$O_2_, and $\overset{\cdot }{V}$CO_2_ and aerobic power (Watt) was defined as the highest average of two consecutive 30 s measurements.

### Familiarization sessions

Familiarization to the specific sessions used in the present study was performed 1 week before the first experimental session. The 6RM load for each exercise in the HS session was defined during the familiarization session for each participant. During the cycling familiarization session, the pedaling resistance applied for the sprints was individually adjusted using an air braked bicycle ergometer (WattBike, WattBike Ltd, Nottingham, UK). This bike was used for both familiarization and experimental trials. To ensure that the participant achieved the highest possible power output during the 8-s sprints at a cadence of 130–140 revolutions per min (RPM) (Hopker et al., 2010), each participant performed at least three sprint at different resistance level with 2 min recovery in between.

### Procedures

All participants were instructed to abstain from strenuous exercise, to perform the same volume of low-intensity training and to have similar diet 48 h before both experimental training sessions, and until the final collection of recovery data 47 h post exercise were performed. All meals, daily activity and sleep were registered for all participants during the data collection period. Prior to both training sessions, participants arrived to the laboratory at the same time following an overnight fast of at least eight h for the baseline blood sample. A standardized breakfast was then served 1.5 h before each training session.

### Body composition

Before breakfast, a direct segmental multi-frequency bioelectrical impedance analysis (DSM-BIA) was performed using the In-Body720 body composition analyzer (Biospace, Tokyo, Japan) to determine body composition. Body mass (kg) and body fat percentage were used in the analyses.

### CMJ and peak cycling power

A continuous 15 min warm-up on a cycle ergometer (Tomahawke IC7, ICG, Germany) at an intensity of 70–80% of HR_max_ was performed before both the CMJ and the peak cycling power test. The CMJ was performed using both legs on a three-dimensional force plate (Kistler 9286B, Kistler Instruments AG, Winterthur, Switzerland) immediately before and 23 and 47 h after each training session. The CMJ started from an upright position and the participants were instructed to descend to a self-chosen depth before jumping vertically with maximal effort. Throughout the CMJ, participants used a hand-on-hips position. Maximum jump height (cm) was calculated using Kistler Measurement, Analysis and Reporting Software (MARS, 2015, S2P, Lubljana, Slovenia). Participants performed at least three jumps, or continued until performance decreased. The best attempt was used in the final analyses.

Peak power (P_peak_) was measured during a 4-s all-out sprint test. The test was completed 23 h after each training session (Herbert et al., 2015; Wainwright et al., 2017). The 4-s all-out test was performed from a standstill start in a seated position, with maximal acceleration from the start, with similar settings as for the SS session. Baseline P_peak_ was defined as the highest peak power during the 4 first seconds obtained in one of the 12 sprint intervals in the SS session.

### HS and SS sessions

HS and SS sessions were performed immediately after the CMJ test. Total duration of the SS and HS sessions, including warm-up, were approximately 45 min.

The HS session consisted of squats with both legs in a smith machine (TKO, Houston, USA), unilateral leg-press (Mobility, Norway), and unilateral hip flexor exercises in cable cross apparatus (Gym 80, Gelsenkirchen, Deutschland), organized as 3 sets of 6 repetition maximum (RM) per exercise, separated by 3 min recovery between sets and 5 min between each exercise. Participants were instructed to carry out the concentric phase with maximal effort, while the eccentric phase was completed as a controlled movement lasting 2 s.

The SS session consisted of three sets of four 8-s intervals with maximal effort, performed from a standstill start in a seated position. Participants started with the individually chosen preferred leg and sitting position in all sprints. Each repetition was separated by 2 min active recovery and each set by 5 min active recovery, consisting of cycling at 70% HR_max_. All SS sessions were completed on a cycle ergometer (WattBike, WattBike Ltd, Nottingham, UK), which allowed measurement of power output (Hopker et al., 2010). The HS and SS sessions were carefully designed to mirror typical training sessions implemented by Norwegian world-class cyclists.

HR was monitored for each set during both HS and SS sessions (Polar V800, Kempele, Finland). In the analyses, session peak HR (HR_peak_) is defined as the mean of the highest obtained HR within each set. RPE was recorded using Borg scale 6–20 (Borg, 1982; Borg et al., 1985) and used as following: immediately after (0 min) the session, 30 min and 1 h post-exercise, the participants were asked the following question: “how exhausted are you in your legs now?” (Day et al., 2004; Wallace et al., 2009). Muscle soreness was measured on a 1–10 scale, 21 and 45 h post-exercise.

### Calculation of power and work

In the HS session, work done (kJ) and power were calculated using the distance and speed of the lifted weights, respectively, by a linear encoder (Muscle Lab, Ergotest Technology, Langesund, Norway) (Bosco et al., 1995). Data were acquired and analyzed using Musclelab software (Musclelab version 8.26 Ergotest Technology). In the squat exercise, 90% of the body weight was added to the external load in the calculation. For the leg-press and hip-flexor exercises, only external load was used. Work done in the eccentric phase was calculated using 1/3 of concentric work (Knuttgen et al., 1982) in all exercises. Average velocity was calculated through the whole range of motion utilized to perform a complete repetition and multiplied by the resistance (in N) to obtain average power (in W) (Bosco et al., 1995). The average power (P_avg_) in each set was calculated as the P_avg_ in each repetition divided by the number of repetitions.

In the SS session, RPM, P_avg_, and peak power (P_peak_) were sampled using Expert software v2.6020 (WattBike Ltd, Nottingham, UK). Work done in each interval was calculated as the P_avg_ during the interval multiplied by the interval duration. Work was calculated for the 12 × 8-s intervals, excluding the low intensity active recovery in-between. Due to missing data in the HS session, work done and power for both sessions are only calculated for 7 cyclists that were representative for the overall performance level of all cyclists (Table 1).

### Blood sampling and processing

Blood samples were collected pre-exercise (at baseline) and post-exercise (5, 30 min, 1, 21, and 45 h). On each occasion, blood was sampled from an antecubital vein into three vacutainers containing K2-EDTA, lithium heparin and clot activator for serum separation (BD Life Sciences, New Jersey). Hematological analyses were performed within 2 h of collection on the K2-EDTA sample. The serum sample was kept at room temperature for approximately 30 min prior to centrifugation at 1,300 × g for 10 min. Serum was then frozen at −80°C until analyses. The lithium heparin samples were stored on ice and centrifuged at 1,800 × g and 4°C for 10 min within 90 min of collection. Plasma was stored at −20°C until analyses.

Serum CK was determined using coupled enzymatic reactions, while serum Mb was measured using a turbidimetric immunoassay, both using the ABX Pentra C400 (Bergman Diagnostica, Horiba Medical, France) according to the manufacturer's protocol. Serum hGH was determined using a solid-phase, two-site chemiluminescent immunometric assay by the IMMULITE 2000 (Siemens Diagnostics, Germany), while plasma lactate was analyzed by the ABX Pentra C400 according to the manufacturer's protocol.

To eliminate inter-assay variance, all samples for a particular assay were thawed once and analyzed in the same assay run. Quality controls for the individual variables were within the acceptable ranges given by manufacturers. All data were corrected for change in plasma volume (Dill and Costill, 1974).

### Statistical analyses

Data are presented as mean ± standard deviation (SD). For serum level of CK, Mb, [La^−^] and hHG, as well as for CMJ performance, DOMS, peak cycling power and maximal RPM obtained in the 4-s all-out sprint test, the repeated measures ANOVA analyses were employed to evaluate main effects of time, training sessions (HS and SS), and the interaction effects between time and sessions. Paired sample *t*-tests were performed for comparison between sessions regarding work done, RPE, session HR_peak_, and session [La^−^] and for post hoc comparisons between and within training sessions for all variables. Statistical significance was determined at an alpha level of <0.05. SPSS® version 24.0 (IBM Corporation, Armonk NY, USA) for Windows® was used for all the statistical analyses.

## Results

### Training load

Total work done (kJ) was about two times larger in the SS session compared to the HS session (*p* < 0.001), with correspondingly higher HR_peak_ (*p* < 0.001) and [La^−^] (*p* < 0.001) (Table 2).

**Table 2:** Baseline and post exercise physiological and perceptual markers of exertion in response to short-sprint interval training (SS session) and heavy strength training (HS session).

	**SS session**	**HS session**
Total Work (kJ)	90.6 ± 2.9	47.9 ± 2.9^*^
RPE immediately post exercise	18.5 ± 1.8	15.9 ± 1.8^*^
RPE 30 min post exercise	10.7 ± 3.9	9.8.±2.3
RPE 1 h post exercise	8.8 ± 3.2	8.6 ± 1.9
HR_peak_ (bpm)	178 ± 10	160 ± 11^*^
[La^−^] at baseline (before breakfast) (mmol/L)	0.9 ± 0.3	0.8 ± 0.5
[La^−^] 5 min post exercise (mmol/L)	14.8 ± 3.1	4.4 ± 1.8^*^
DOMS 21 h post exercise (1−10)	1.5 ± 1.2	2.1 ± 1.7
DOMS 45 h post exercise (1−10)	1.1 ± 0.3	2.5 ± 1.5^*^
CMJ at baseline (cm)	34.0 ± 5.8	
CMJ 23 h post exercise (cm)	33.2 ± 5.4	34.0 ± 5.1
CMJ 47 h post exercise (cm)	34.0 ± 5.8	34.0 ± 4.2
Peak cycling power (W) at baseline	1,183 ± 98	
Peak cycling power (W) 23 h post exercise	1, 215 ± 128	1, 212 ± 108
Peak RPM at baseline	140 ± 4	
Peak RPM 23 h post exercise	141 ± 5	142 ± 5

*RPE, rating of perceived exertion (6–20); [La^−^], plasma blood lactate concentration; DOMS, delayed onset muscle soreness; CMJ, counter movement jump; RPM, revolutions per minute. Session HR_peak_, average heart rate values from all intervals and reps in HS and SS session. Peak cycling power and peak RPM at baseline are maximal values obtained during the first 4 s in an 8-s maximal cycling sprint. Peak cycling power and peak RPM 23 h post exercise are maximal values obtained in a 4-s all-out sprint test*.

* *Significant difference between SS and HS session (p < 0.05)*.

### Short-sprint training session

P_peak_ and P_avg_ for the intervals in the entire SS session were 1113 ± 102 and 964 ± 75 W, respectively. From set 1 (sprint 1–4) to set 3 (sprint 9–12) there was a 5.3% decrease in P_peak_ (1,142 ± 103, and 1,082 ± 113 W, *p* = 0.030) and a non-significant 3.0% decrease in P_avg_ (982 ± 76, and 951 ± 77 W, *p* = 0.123), with stable P_peak_ and P_avg_ within sets. There was an increase in [La^−^] from set 1 to 2 (*p* = 0.003) and from set 1 to set 3 (*p* < 0.001) (10.9 ± 1.9, 12.6 ± 2.4, and 13.6 ± 1.9 mmol·L^−1^, respectively).

### Heavy strength training session

P_avg_ in the entire sessions of squat, hip flexion and leg press were 321 ± 35, 438 ± 53, and 250 ± 53 W, respectively. There was a decrease of 8.9% in P_avg_ from set 1 to 3 in squat (337 ± 40 and 307 ± 37 W, respectively, *p* = 0.003), an increase of 11.2% from set 1 to 3 in hip flexion (410 ± 55, and 456 ± 53 W, respectively, *p* = 0.030) and of 12.0% in leg press (234 ± 51, and 262 ± 61 W, respectively, *p* = 0.046). There was a significant increase in [La^−^] during the HS session from first to last exercise (*p* = 0.010) (4.8 ± 1.0, 6.1 ± 2.8 and 6.0 ± 1.6 mmol·L^−1^ respectively).

### Power production and perceptual responses

There were no differences in CMJ height (23 and 47 h post exercise), nor in P_peak_ or RPM obtained in the 4-s all out sprint test (23 h post exercise) between the HS and SS sessions, and no changes from baseline values for any of the sessions (Table 2). For RPE, there was a main effect of time (0 min, 30 min and 1 h post exercise) (*p* < 0.001), and an interaction between time and session (*p* = 0.022) with higher RPE (*p* = 0.010) immediately after the SS session compared to the HS session. There was a main effect of session for DOMS (*p* = 0.043), with a significantly higher DOMS score 45 h after the HS session compared to the SS session (*p* = 0.010), with similar levels after 21 h.

### Biochemical markers

**Figure 1:**
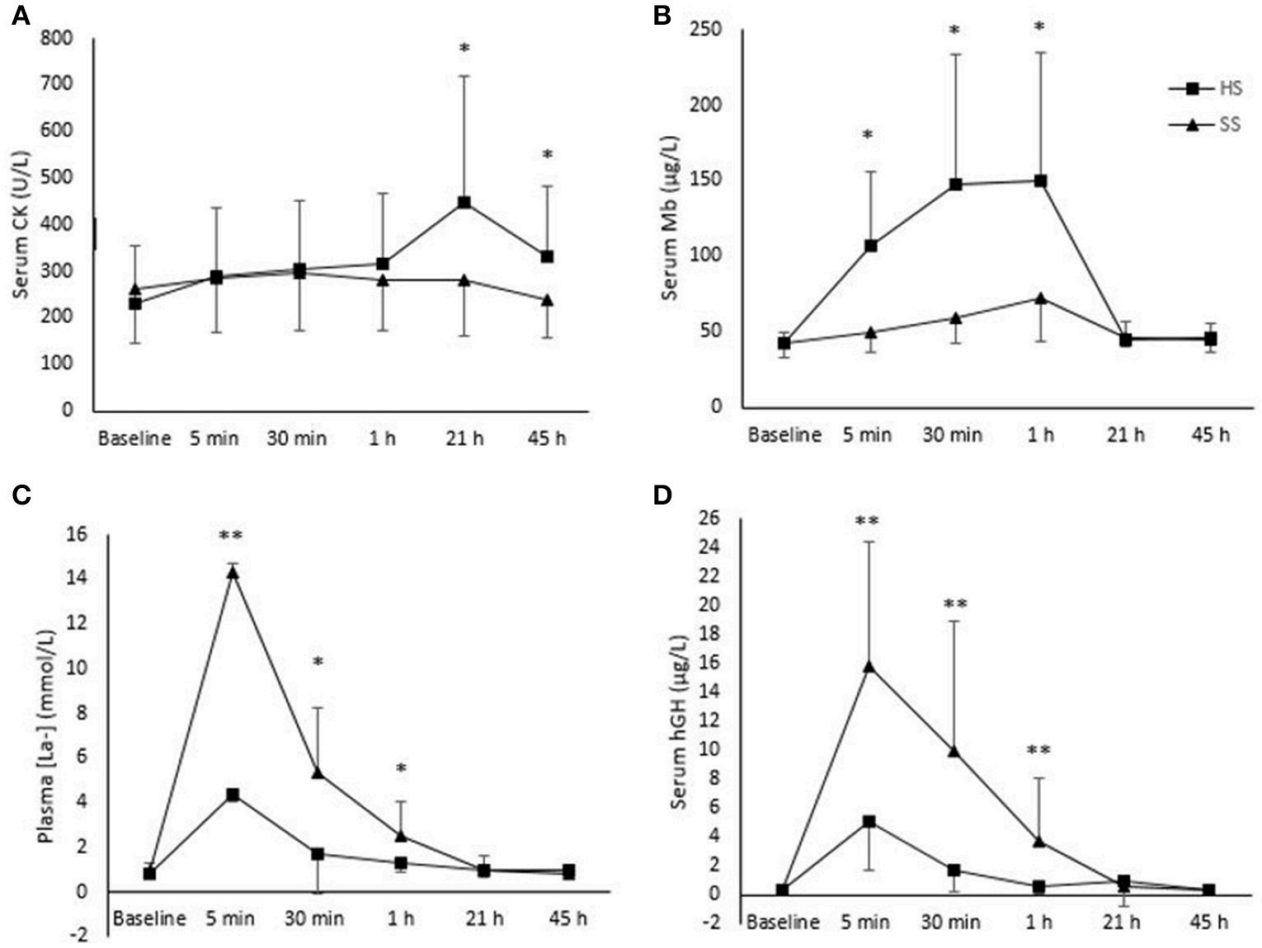
Changes in: **(A)** creatinine kinase (CK), **(B)** myoglobin (Mb), **(C)** lactate concentration ([La^−^]) and **(D)** human growth hormone (hGH) following a heavy strength training session (HS) and a short-sprint training session (SS) pre- (baseline) and post-exercise (5 min, 30 min, 1 h and 21 and 45 h) in 11 well-trained cyclists. Difference between sessions are indicated as ^*^p < 0.05, ^**^p < 0.001. Vertical lines represent standard deviation.

There were main effects of time for CK (*p* = 0.001), Mb (*p* < 0.001), hGH (*p* < 0.001) and [La^−^] (*p* < 0.001), and main effects of session for Mb (with higher levels for HS than SS; *p* = 0.004), for hGH (with higher levels for SS than HS; *p* = 0.003) and for [La^−^] (with higher levels for SS than HS; *p* < 0.001). Interaction effects of time × session were found for the levels of CK (*p* = 0.002), Mb, hGH, and [La^−^] (all *p* < 0.001 Figure 1). Higher levels of CK and Mb were reported following the HS session, whereas higher levels of hGH and [La^−^] were detected following the SS session.

### Creatine kinase

CK was higher following the HS compared to the SS session both 21 h (*p* = 0.023) and 45 h (*p* = 0.38) post exercise (Figure 1A). Moreover, increased CK from baseline was seen 5, 30 min, 1, 21, and 45 h after completing the HS session (*p* < 0.001, *p* < 0.001, *p* < 0.001, *p* = 0.015, *p* = 0.034, respectively). An increase in CK from baseline was only seen 5 min and 30 min after completing the SS session (*p* = 0.002 and *p* = 0.007, respectively), while no difference was seen 1, 21, and 45 h post session (Figure 1A).

### Myoglobin

Mb was higher following the HS compared to the SS session 5 min, 30 min, and 1 h post exercise (*p* = 0.002, *p* = 0.004, and 0 = 0.005, respectively; Figure 1B). Increased Mb from baseline was seen 5 min, 30 min and 1 h both after completing the HS (*p* = 0.002, *p* = 0.003, and *p* = 0.002 respectively) and the SS session (*p* = 0.009, *p* = 0.001, and *p* = 0.003, respectively). No difference from baseline was seen 21 and 45 h post in any of the sessions (Figure 1B).

### Lactate

[La^−^] was higher following the SS compared to the HS session 5 min, 30 min, and 1 h post exercise (*p* < 0.001 for all comparisons) (Figure 1C). An increase in [La^−^] from baseline was seen 5 min, 30 min and 1 h after completing the HS and the SS session (*p* < 0.001 for all comparisons), while no differences were seen 21 and 45 h post sessions (Figure 1C).

### Human growth hormone

Level of hGH was higher following the SS compared to the HS session 5 min, 30 min and 1 h post exercise (*p* = <0.001, *p* = 0.006, and *p* = 0.019, respectively; Figure 1D). An increase in hGH from baseline was seen 5 min post exercise following both the HS (*p* = 0.016) and the SS (*p* = 0.002) (Figure 1D).

## Discussion

The primary aim of the current study was to compare power production and biochemical markers of metabolic stress and muscle damage following a HS and a SS training session designed to mirror typical training sessions implemented by world-class cyclists. A main result was higher levels of [La^−^] and hGH 5 min, 30 min and 1 h following the SS session compared to the HS session, as well as higher levels of Mb following the HS session compared to the SS session at the same time points. However, no differences between sessions were found 21 h post exercise. As expected, the serum level of CK was higher 21 and 45 h following the HS session compared to the SS session. In addition, DOMS was higher 45 h after the HS session compared to the SS session. There was no difference in CMJ performances 23 or 47 h post exercise, or in P_peak_ and RPM obtained in the 4-s all-out sprint test 23 h post exercise between the HS and SS sessions.

The inherent differences in load exerted from the HS and SS sessions led to subsequent diversities in the acute responses, which is in line with previously published studies in this area (Kraemer et al., 1990; Godfrey et al., 2003; Mougios, 2007; Speranza et al., 2007; Bishop et al., 2008; Coffey et al., 2009; Brancaccio et al., 2010; Koch et al., 2014; Bessa et al., 2016; Soares and Bozza, 2016). However, while none of the previous studies compared the acute responses between sprint and strength training, the novelty of our approach were the paired samples design used to compare acute responses and recovery rates following typical training sessions. In addition, we used a complex battery of biochemical markers indicating metabolic stress and muscle damage among highly trained cyclists. The acute responses following these sessions seemed to be influenced both by the total work done that was larger for the SS session and the peak and average power/load that was higher during the HS session. Specifically, the work done during the twelve 8-s maximal cycling sprints was about 2 times larger compared to the HS training session containing three sets of 6RM using three different strength exercises. This was reflected in higher RPE scores, HR_peak_ and levels of [La^−^] following the SS compared to the HS session. In comparisons, the higher levels of [La^−^] and hGH following the SS session indicate larger metabolic disturbances than for the HS session (Smilios et al., 2003; Gladden, 2004; Stokes et al., 2004). The increase of hGH after high-intensity exercises are well recognized (Godfrey et al., 2003), since greater demands of anaerobic glycolysis stimulates serum hGH elevations (Kraemer et al., 1990). Overall, the higher levels of [La^−^] and hGH 5 min, 30 min and 1 h after the SS session compared to the HS session, coincide with the larger work done and most likely reflects higher metabolic stress than the HS session. These differences in recovery rates between sessions provide new insight that might help coaches and athletes to understand the load and recovery rates from such sessions. In this case, these markers remained back to baseline within 1 day after both sessions, indicating that well-trained cyclists are metabolically recovered and can train as normal on the subsequent day.

Furthermore, the higher levels of Mb until 1 h post exercise and greater CK levels 21 and 45 h post exercise reported after the HS session compared to the SS session indicates larger muscle damage (Mougios, 2007; Speranza et al., 2007; Bishop et al., 2008; Brancaccio et al., 2010; Koch et al., 2014; Bessa et al., 2016; Soares and Bozza, 2016). It has previously been shown that weight bearing exercises, including eccentric muscle actions, cause the highest increase in serum level of CK (Brancaccio et al., 2007, 2010; Koch et al., 2014) and Mb (Speranza et al., 2007; Soares and Bozza, 2016). In the present study, exercises in the HS session were performed with slow movements in the eccentric phase and with maximal effort and movement velocity in the concentric phase, while the SS session was performed with lower resistance and less eccentric action. This difference may have led to more muscle damage for the HS session, which subsequently requires longer time to be fully recovered—an important and novel finding to be aware of when implementing strength training in well-trained cyclists' training schedule.

In the present study, a relatively low DOMS score was reported after both sessions, although there was a significantly higher score after the HS session compared to the SS session 45 h post session. Our findings are generally in line with several previous studies where DOMS was increased 24–72 h following a strength training session (Armstrong, 1984; Nosaka et al., 2002; Kraemer and Ratamess, 2004; Bishop et al., 2008). However, the relatively low DOMS scores in the present study might be due to the higher fitness levels of our participants, as well as their high level of familiarization to such sessions. Differences in both acute responses and recovery rates between athletes of different training levels or with various degrees of familiarization are important distinctions to be aware of when comparing studies. However, the use of CK levels and DOMS score as measures of recovery is controversial (Nosaka et al., 2002), and previous studies show no correlation between changes in CK levels, DOMS scores and performance tests measuring force production following fatiguing events (Byrne et al., 2004). This also applies to our data, where the development of CK, DOMS and power production in CMJ and 4-s all-out sprints follows different patterns.

According to Paulsen et al. (2012), force production during performance tests are essential measures of recovery status. Here we measured CMJ height and P_peak_ on a cycle ergometer, which reflects the ability to produce force and power, and should provide valid measures of recovery status in that context. However, we found no difference between the HS and SS session in either of these measures, which are likely explained by the higher fitness levels and familiarization of our cyclists compared to previous studies. Furthermore, no difference from baseline values was found neither after the HS nor after the SS session. This is in contrast to the decline in force production previously reported both after HS and SS training (Raastad and Hallen, 2000; Andersson et al., 2008; Haugvad et al., 2014; Gathercole et al., 2015). Although the relatively rapid recovery among our participants might be due to their high fitness level, we cannot exclude that endurance athletes may have limited ability to produce force and power, and thereby induce less muscle damage compared to studies done on power-trained athlete groups. Less muscle damage in our group may also be influenced by our inclusion criterion that that HS and SS training should have been part of the cyclists' weekly training before entering our study.

## Conclusion

Our findings indicate that SS training provides greater metabolic stress than HS training, whereas HS training leads to more muscle damage compared to that caused by SS training. However, although higher CK level and muscle soreness were present 45 h post the HS training session the ability to produce power remained back to baseline already 23 h after both training sessions indicating a rapid rate of recovery in our well-trained cyclists.

### Practical application

Based on our findings, it appears that sprint training provides a different type of response than strength training among well-trained cyclists, with higher metabolic disturbances after SS training and greater muscular damage subsequent to HS training. This must be taken into account by coaches and athletes when including such sessions in the weekly training plans. However, it seems like both types of training require relatively short recovery times compared to previous studies on less trained participants. Such sessions will therefore not substantially influence sessions performed approximately 24 h later in well-trained cyclist who are already familiar with sprint and strength training. Still, there seems to be indications of muscle damage and perceptual feelings of muscle fatigue the first 45 h after HS training that athletes/coaches should be aware of.

## Ethics statement

This study was carried out in accordance with the recommendations of The Norwegian Data protection Authority with written informed consent from all subjects. All subjects gave written informed consent in accordance with the Declaration of Helsinki. The protocol was approved by the The Norwegian Data protection (45048).

## Author contributions

MK: Planning, data collection, analyzing, and writing; ØS, ET, GP, and BR: Planning, analyzing, and writing; IS: Analyzing and writing; EE, IN, KR, and AR: Blood data collection, analyzing, writing; VI and KS: Data collection and analyzing; HG: Data collection, analyzing, and writing.

### Conflict of interest statement

The authors declare that the research was conducted in the absence of any commercial or financial relationships that could be construed as a potential conflict of interest.
